# Supplementary material for: LongStitch: high-quality genome assembly correction and scaffolding using long reads
Source: BMC Bioinformatics. 2021 Oct 30;22:534. doi: 10.1186/s12859-021-04451-7 (PMC8557608; doi:10.1186/s12859-021-04451-7)
Supplement: Supplementary file 1 — Additional file 1. Table S1. Sequencing data used for the correction and scaffolding runs. Table S2. Baseline assemblies used for correction and scaffolding runs. Table S3. Availability of baseline assemblies used for correction and scaffolding runs. Table S4. Reference genome builds used for QUAST assembly analysis. Table S5. Contiguity, correctness and benchmarking statistics for running the default steps of LongStitch (up to ntLink) on human assemblies. Table S6. Contiguity, correctness and benchmarking statistics for running LRScaf on human assemblies. Table S7. Contiguity, correctness and benchmarking statistics for running OPERA-LG on human assemblies. Table S8. Contiguity, correctness and benchmarking statistics for running LongStitch, LRScaf and OPERA-LG on C. elegans assemblies. Table S9. Contiguity, correctness and benchmarking statistics for running LongStitch, LRScaf and OPERA-LG on O. sativa assemblies. Table S10. Positive predictive value (PPV) and true positive rate (TPR) of Tigmint-long. Table S11. Contiguity, correctness and benchmarking statistics for running LongStitch including the optional the ARKS-long step on human assemblies. Table S12. Summarizing the number of matching minimizers per ntLink-joined contig pair. Figure S1. Schematic describing gap size estimation algorithm in ntLink. Figure S2. Contiguity and correctness statistics of assemblies improved using LRScaf with different parameter combinations and LongStitch. Figure S3. Jupiter consistency plots showing the contiguity and correctness of LongStitch and LRScaf assemblies. Figure S4. Benchmarking results of improving assemblies with LongStitch, LRScaf or OPERA-LG. Figure S5. Contiguity and correctness results for each step the LongStitch pipeline, including the optional ARKS-long step, LRScaf and OPERA-LG on human assemblies. Figure S6. Benchmarking results for LRScaf and the LongStitch pipeline including the optional ARKS-long step on human assemblies. Figure S7. Time breakdown for e [file 12859_2021_4451_MOESM1_ESM.pdf]

LongStitch – High-quality genome assembly correction and scaffolding using long reads

Lauren Coombe*, Janet X Li*, Theodora Lo*, Johnathan Wong, Vladimir Nikolic, René L Warren, Inanc Birol
* Joint first authors

Additional File 1

Table of Contents

Table S1. Sequencing data used for the correction and scaffolding runs. 3
Table S2. Baseline assemblies used for correction and scaffolding runs. 4
Table S3. Availability of baseline assemblies used for correction and scaffolding runs. 5
Table S4. Reference genome builds used for QUAST assembly analysis......................................6
Table S5. Contiguity, correctness and benchmarking statistics for running the default steps of LongStitch (up to ntLink) on human assemblies. 6
Table S6. Contiguity, correctness and benchmarking statistics for running LRScaf on human assemblies. 7
Table S7. Contiguity, correctness and benchmarking statistics for running OPERA-LG on human assemblies. 7
Table S8. Contiguity, correctness and benchmarking statistics for running LongStitch, LRScaf and OPERA-LG on *C. elegans* assemblies..8
Table S9. Contiguity, correctness and benchmarking statistics for running LongStitch, LRScaf and OPERA-LG on *O. sativa* assemblies. 9
Table S10. Positive predictive value (PPV) and true positive rate (TPR) of Tigmint-long............10
Table S11. Contiguity, correctness and benchmarking statistics for running LongStitch including the optional ARKS-long step on human assemblies 11
Table S12. Summarizing the number of matching minimizers per ntLink-joined contig pair. 11
Figure S1. Schematic describing the gap size estimation algorithm in ntLink. 12
Figure S2. Contiguity and correctness statistics of assemblies improved using LRScaf with different parameter combinations and LongStitch. 13
Figure S3. Jupiter consistency plots showing the contiguity and correctness of LongStitch and LRScaf assemblies. 14

Figure S4. Benchmarking results of improving assemblies with LongStitch, LRScaf or OPERA-LG. 16
Figure S5. Contiguity and correctness results for running each step of the LongStitch pipeline, including the optional ARKS-long step, LRScaf and OPERA-LG on human assemblies. 17
Figure S6. Benchmarking results for running LRScaf and the LongStitch pipeline including the optional ARKS-long step on human assemblies. 18
Figure S7. Time breakdown for each of the steps in LongStitch, including the optional ARKS-long step, and LRScaf. 19
Figure S8. Contiguity and correctness results from sweeping on the ntLink *k* and *w* parameters for the default steps of LongStitch. 20
Figure S9. Benchmarking results from sweeping on the ntLink *k* and *w* parameters for the default steps of LongStitch. 21

Supplementary Tables

Table S1. Sequencing data used for the correction and scaffolding runs.

Species	Individual/ Strain	Reads type	Fold coverage	Platform	Accession(s)/Source
H. sapiens	NA12878	Short	54	Illumina HiSeq	Illumina basespace – run title: “HiSeq 2500: TruSeq PCR-Free DNA 2x251 (NA12878)”
H. sapiens	NA12878	MPET	N/A	Illumina HiSeq	ERR262997
H. sapiens	NA12878	Long	39	ONT	SRR10965087
H. sapiens	NA19240	Short	62	Illumina HiSeq	SRR3189758, SRR3189759
H. sapiens	NA19240	Long	49	ONT	ERR3219854, ERR3219857
H. sapiens	NA24385	Short	58	Illumina HiSeq	SRR11321732
H. sapiens	NA24385	Long	51	ONT	s3://ont-open-data/gm24385_2020.11/analysis/r9.4.1/20201026_1644_2-E5-H5_PAG07162_d7f262d5/guppy_v4.0.11_r9.4.1_hac_prom/basecalls.fastq.gz
C. elegans	N2	Short	75	Illumina Genome Analyzer IIx	DRR008444
C. elegans	N2	Long	40	ONT	ERR2092776 (randomly subsampled to 40x)
O. sativa	Japonica group	Short	67	Illumina NovaSeq	SRR10567117
O. sativa	Japonica group	Long	63	ONT	SRR10589512-SRR10589711

ONT: Oxford Nanopore Technologies
MPET: Mate-pair sequencing reads

Table S2. Baseline assemblies used for correction and scaffolding runs. 'n' is the number of contigs over 3 kbp. Default parameters were used for the assemblers, with the exception of the parameters listed.

Species	Individual	Assembly reads	Assembler	Parameters	n	NG50 (bp)	NGA50 (bp)	Largest contig (bp)	Number of extensive misassemblies	Local misassemblies
H. sapiens	NA12878	Short + MPET	ABySS 2.1.5	k=128, kc=3, B=115G, j=24	6,656	1,175,297	1,131,267	7,807,150	462	7,589
H. sapiens	NA12878	Long	Shasta 0.5.1	--threads 48	3,596	2,617,486	2,346,570	17,057,497	533	5,184
H. sapiens	NA19240	Short	ABySS 2.2.3	k=112, kc=3, B=150G, j=48, N=5-20	190,813	14,618	14,512	202,526	969	951
H. sapiens	NA19240	Long	Shasta 0.5.1	--threads 48	4,316	2,389,148	2,213,238	13,988,698	526	6,516
H. sapiens	NA24385	Short	ABySS 2.2.3	k=112, kc=3, B=150G, j=48, N=5-20	44,555	102,102	97,340	1,078,380	2,024	1,077
H. sapiens	NA24385	Long	Shasta 0.5.1	--threads 48	4,268	5,734,421	4,876,524	30,135,269	768	4,054
C. elegans	N2 strain	Short	ABySS 2.1.4	k=64, kc=3, B=10G, j=8	4552	30,293	27,037	339,951	448	89
C. elegans	N2 strain	Long	Shasta 0.5.1	--threads 48	299	1,385,395	783,719	5,326,010	69	1,021
O. sativa	Japonica group	Short	ABySS 2.2.3	k=112, kc=2, B=50G, j=48	16,257	22,852	20,545	225,597	1,407	1,015
O. sativa	Japonica group	Long	Canu* (GCA_002573525.1)	N/A	588	2,733,017	1,173,940	10,356,529	2,424	2,700

***** Downloaded from NCBI with specified accession. To generate assembly, Nie, SJ, *et al* (2017) assembled PacBio reads using Canu, then polished the assembly using Pilon.

Table S3. Availability of baseline assemblies used for correction and scaffolding runs.

Species	Individual/ Strain	Assembly reads	Assembler	URL
H. sapiens	NA12878	Short + MPET	ABySS 2.1.5	https://www.bcgsc.ca/downloads/btl/LongStitch/baseline_assemblies/NA12878/abyss/NA12878.abyss-mpet.fa.gz
H. sapiens	NA12878	Long	Shasta 0.5.1	https://www.bcgsc.ca/downloads/btl/LongStitch/baseline_assemblies/NA12878/shasta/NA12878.shasta-polished.fa.gz
H. sapiens	NA19240	Short	ABySS 2.2.3	https://www.bcgsc.ca/downloads/btl/LongStitch/baseline_assemblies/NA19240/abyss/NA19240.abyss.fa.gz
H. sapiens	NA19240	Long	Shasta 0.5.1	https://www.bcgsc.ca/downloads/btl/LongStitch/baseline_assemblies/NA19240/shasta/NA19240.shasta-polished.fa.gz
H. sapiens	NA24385	Short	ABySS 2.2.3	https://www.bcgsc.ca/downloads/btl/LongStitch/baseline_assemblies/NA24385/abyss/NA24385.abyss.fa.gz
H. sapiens	NA24385	Long	Shasta 0.5.1	https://www.bcgsc.ca/downloads/btl/LongStitch/baseline_assemblies/NA24385/shasta/NA24385.shasta-polished.fa.gz
C. elegans	N2	Short	ABySS 2.1.4	https://www.bcgsc.ca/downloads/btl/LongStitch/baseline_assemblies/celegans/abyss/celegans.abyss.fa.gz
C. elegans	N2	Long	Shasta 0.5.1	https://www.bcgsc.ca/downloads/btl/LongStitch/baseline_assemblies/celegans/shasta/celegans.shasta-polished.fa.gz
O. sativa	Japonica group	Short	ABySS 2.2.3	https://www.bcgsc.ca/downloads/btl/LongStitch/baseline_assemblies/osativa/abyss/osativa.abyss.fa.gz
O. sativa	Japonica group	Long	Canu	https://www.ncbi.nlm.nih.gov/assembly/GCA_002573525.1/

Table S4. Reference genome builds used for QUAST assembly analysis.

Species	Reference genome build	Reference genome accession
H. sapiens	GRCh38	GCA_000001405.15
C. elegans	Bristol N2	GCF_000002985.6
O. sativa	IRGSP-1.0	GCF_001433935.1

Table S5. Contiguity, correctness and benchmarking statistics for running the default steps of LongStitch (up to ntLink) on human assemblies. Default parameters were used, with the exception of the ntLink parameters listed. Misassemblies were assessed using QUAST. 'n' is the number of contigs over 3 kbp. The fold increase of extensive misassemblies (as defined by QUAST) is relative to the baseline.

Individual	Assembler	ntLink parameters	n	NG50 (Mbp)	NGA50 (Mbp)	Number of extensive misassemblies	Fold increase of extensive misassemblies	Relocations	Inversions	Translocations	Local misassemblies	Time (h)	Peak memory (GB)
NA12878	ABySS	k=32, w=100	2,521	15.32	12.19	593	1.28	375	25	193	7,516	3.37	18.95
NA12878	Shasta	k=24, w=250	3,126	5.70	4.68	600	1.13	407	27	166	5,267	3.00	17.44
NA19240	ABySS	k=40, w=100	5,437	5.11	4.42	500	0.52	212	32	256	961	4.34	19.33
NA19240	Shasta	k=24, w=250	2,571	18.66	12.93	622	1.18	390	35	197	6,432	3.65	17.09
NA24385	ABySS	k=40, w=500	5,375	8.12	6.66	745	0.37	415	39	291	1,216	4.29	22.44
NA24385	Shasta	k=40, w=500	3,440	26.97	16.61	892	1.16	588	40	264	4,020	4.48	19.31

Table S6. Contiguity, correctness and benchmarking statistics for running LRScaf on human assemblies. Misassemblies were assessed using QUAST. 'n' is the number of contigs over 3 kbp. The fold increase of extensive misassemblies is relative to the baseline.

Individual	Assembler	n	NG50 (Mbp)	NGA50 (Mbp)	Number of extensive misassemblies	Fold increase of extensive misassemblies	Relocations	Inversions	Translocations	Local misassemblies	Time (h)	Peak memory (GB)
NA12878	ABySS	2,525	11.49	8.66	1,021	2.21	581	33	407	8,207	2.71	21.45
NA12878	Shasta	1,711	8.51	6.61	982	1.84	654	37	291	5,983	3.46	18.52
NA19240	ABySS	3,079	3.10	1.96	1,871	1.93	560	32	1,279	1,499	44.61	21.29
NA19240	Shasta	2,068	12.40	9.48	910	1.73	559	42	309	7,158	3.70	17.72
NA24385	ABySS	4,015	2.23	1.30	2,769	1.37	831	56	1,882	1,245	5.89	23.54
NA24385	Shasta	3,030	24.28	16.61	1,426	1.86	859	55	512	4,739	13.03	19.28

Table S7. Contiguity, correctness and benchmarking statistics for running OPERA-LG on human assemblies. Misassemblies were assessed using QUAST. 'n' is the number of contigs over 3 kbp. The fold increase of extensive misassemblies is relative to the baseline.

Individual	Assembler	n	NG50 (Mbp)	NGA50 (Mbp)	Number of extensive misassemblies	Fold increase of extensive misassemblies	Relocations	Inversions	Translocations	Local misassemblies	Time (h)	Peak memory (GB)
NA12878	ABySS	7,633	11.57	4.38	4,168	9.02	1,955	68	2,145	8,357	226.86	284.37
NA12878	Shasta	2,650	5.70	4.63	611	1.15	416	29	166	5,201	273.41	290.70
NA19240	ABySS	6,062	1.83	0.95	3,877	4.00	1,586	49	2,242	1,721	347.78	149.26
NA19240	Shasta	3,421	3.94	3.35	636	1.21	351	38	247	6,467	300.75	138.88
NA24385	ABySS	7,332	0.95	0.57	4,970	2.46	1,865	74	3,031	2,020	254.69	166.61
NA24385	Shasta	3,904	8.20	6.57	1,169	1.52	685	44	440	4,129	307.16	181.04

Table S8. Contiguity, correctness and benchmarking statistics for running LongStitch, LRScaf and OPERA-LG on *C. elegans* assemblies. Misassemblies were assessed using QUAST. ‘n’ is the number of contigs over 3 kbp. The fold increase of extensive misassemblies is relative to the baseline.

Tool	Assembler	n	NG50 (kbp)	NGA50 (kbp)	Number of extensive misassemblies	Fold increase of extensive misassemblies	Relocations	Inversions	Translocations	Local misassemblies	Time (min)	Peak memory (GB)
LongStitch (default)	ABySS	936	598.70	437.23	176	0.39	57	30	89	90	4.27	2.65
LongStitch (to ARKS-long)	ABySS	756	971.83	568.50	196	0.44	66	38	92	90	12.02	2.65
LRScaf	ABySS	558	390.40	247.45	499	1.11	132	45	322	137	2.08	4.51
OPERA-LG	ABySS	523	397.34	173.89	576	1.29	155	47	374	159	215.55	16.24
LongStitch (default)	Shasta	239	2,119.26	961.86	67	0.97	55	5	7	1,022	4.28	2.94
LongStitch (to ARKS-long)	Shasta	218	2,513.58	1,139.36	79	1.14	60	5	14	1,040	11.35	2.94
LRScaf	Shasta	181	2,665.80	1,139.36	75	1.09	62	6	7	1,103	0.19	2.25
OPERA-LG	Shasta	233	1,897.01	937.18	75	1.09	60	6	9	1,052	407.15	15.78

- ntLink parameters: ABySS – k=40, w=100; Shasta – k=24, w=250

Table S9. Contiguity, correctness and benchmarking statistics for running LongStitch, LRScaf and OPERA-LG on *O. sativa* assemblies. Misassemblies were assessed using QUAST. 'n' is the number of contigs over 3 kbp. The fold increase of extensive misassemblies is relative to the baseline.

Tool	Assembler	n	NG50 (kbp)	NGA50 (kbp)	Number of extensive misassemblies	Fold increase of extensive misassemblies	Relocations	Inversions	Translocations	Local misassemblies	Time (min)	Peak memory (GB)
LongStitch (default)	ABySS	3,129	803.38	375.70	890	0.63	402	21	467	1,209	24.85	4.38
LongStitch (to ARKS-long)	ABySS	2,665	1,225.85	460.95	1,029	0.73	450	59	520	1,225	78.17	4.38
LRScaf	ABySS	1,863	558.98	241.39	1,967	1.40	586	26	1,355	1,411	24.39	11.10
OPERA-LG	ABySS	1,557	577.14	137.03	3,224	2.29	801	25	2,398	1,571	973.62	24.42
LongStitch (default)	Canu*	790	5,899.99	1,683.26	2,186	0.90	695	54	1,437	2,649	26.03	5.28
LongStitch (to ARKS-long)	Canu*	763	7,377.78	1,644.25	2,213	0.91	700	54	1,459	2,651	68.26	5.28
LRScaf	Canu*	515	3,961.40	1,483.04	2,542	1.05	760	50	1,732	2,835	14.94	4.62
OPERA-LG	Canu*	507	3,426.10	1,402.49	2,449	1.01	742	49	1,658	2,711	1,826.13	39.26

* NCBI accession: GCA_002573525.1

- ntLink parameters: ABySS – k=32, w=100; Canu: k=24, w=500

Table S10. Positive predictive value (PPV) and true positive rate (TPR) of Tigmint-long. PPV was calculated as corrected misassemblies / total attempted corrections, and TPR was calculated as corrected misassemblies / baseline misassemblies. “Misassemblies” is the sum of extensive and local misassemblies reported by QUAST, and attempted corrections are counted and logged during the Tigmint-cut stage of Tigmint-long.

Species	Individual	Assembler	Number of baseline misassemblies	Number of misassemblies after Tigmint-long	Number of corrected misassemblies	Number of attempted corrections	PPV	TPR
H. sapiens	NA12878	ABySS	8,051	7,914	137	772	0.177	0.017
H. sapiens	NA12878	Shasta	5,717	5,523	194	1,226	0.158	0.034
H. sapiens	NA19240	ABySS	1,920	1,269	651	680	0.957	0.339
H. sapiens	NA19240	Shasta	7,042	7,008	34	64	0.531	0.005
H. sapiens	NA24385	ABySS	3,101	1,717	1,384	1,557	0.889	0.446
H. sapiens	NA24385	Shasta	4,822	4,816	6	169	0.036	0.001
C. elegans	N2 strain	ABySS	533	258	275	290	0.948	0.516
C. elegans	N2 strain	Shasta	1,077	1,072	5	24	0.208	0.005
O. sativa	Japonica group	ABySS	2,410	1,868	542	576	0.941	0.225
O. sativa	Japonica group	Canu	5,051	4,736	315	320	0.984	0.062

Table S11. Contiguity, correctness and benchmarking statistics for running LongStitch including the optional ARKS-long step on human assemblies. Default parameters were used, with the exception of the ntLink parameters listed. Misassemblies were assessed using QUAST. 'n' is the number of contigs over 3 kbp. The fold increase of extensive misassemblies is relative to the baseline.

Individual	Assembler	ntLink parameters	n	NG50 (Mbp)	NGA50 (Mbp)	Number of extensive misassemblies	Fold increase of extensive misassemblies	Relocations	Inversions	Translocations	Local misassemblies	Time (h)	Peak memory (GB)
NA12878	ABySS	k=32, w=100	2,135	20.89	14.12	746	1.61	488	29	229	7,522	6.76	18.95
NA12878	Shasta	k=24, w=250	2,702	6.27	5.08	752	1.41	499	35	218	5,353	6.46	17.44
NA19240	ABySS	k=40, w=100	4,359	10.94	6.94	626	0.65	312	44	270	961	9.07	19.33
NA19240	Shasta	k=24, w=250	2,120	23.38	13.71	773	1.47	470	39	264	6,496	8.13	17.09
NA24385	ABySS	k=40, w=500	4,453	13.88	9.15	1,003	0.50	570	93	340	1,226	9.59	22.44
NA24385	Shasta	k=40, w=500	3,049	32.20	16.66	969	1.26	638	48	283	4,016	9.70	19.31

Table S12. Summarizing the number of matching minimizers per ntLink-joined contig pair.

Species	Individual	Assembly	ntLink parameters	Minimum	25th percentile	Median	75th percentile	Maximum
H. sapiens	NA12878	short	k=32, w=100	2	5	11	25	251
H. sapiens	NA12878	long	k=24, w=250	2	3	9	19	136
H. sapiens	NA19240	short	k=40, w=100	2	7	13	22	158
H. sapiens	NA19240	long	k=24, w=250	2	4	11	19	85
H. sapiens	NA24385	short	k=40, w=500	2	11	20	32	169
H. sapiens	NA24385	long	k=40, w=500	2	4	13	27	153
C. elegans	N2 strain	short	k=40, w=100	2	5	8	14	94
C. elegans	N2 strain	long	k=24, w=250	2	3	7	13	69
O. sativa	Japonica group	short	k=32, w=100	2	7	13	23	223
O. sativa	Japonica group	long	k=24, w=500	2	3	6	9	47

Supplementary Figures

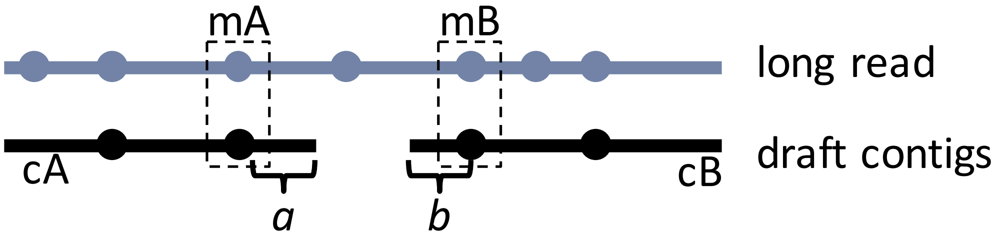

$$gap\ size = |pos(mA, long\_read) - pos(mB, long\_read)| - a - b$$

where $a = distance\ from\ pos(mA, draft)\ to\ end\ of\ cA$

and $b = distance\ from\ pos(mB, draft)\ to\ end\ of\ cB$

Figure S1. Schematic describing the gap size estimation algorithm in ntLink.

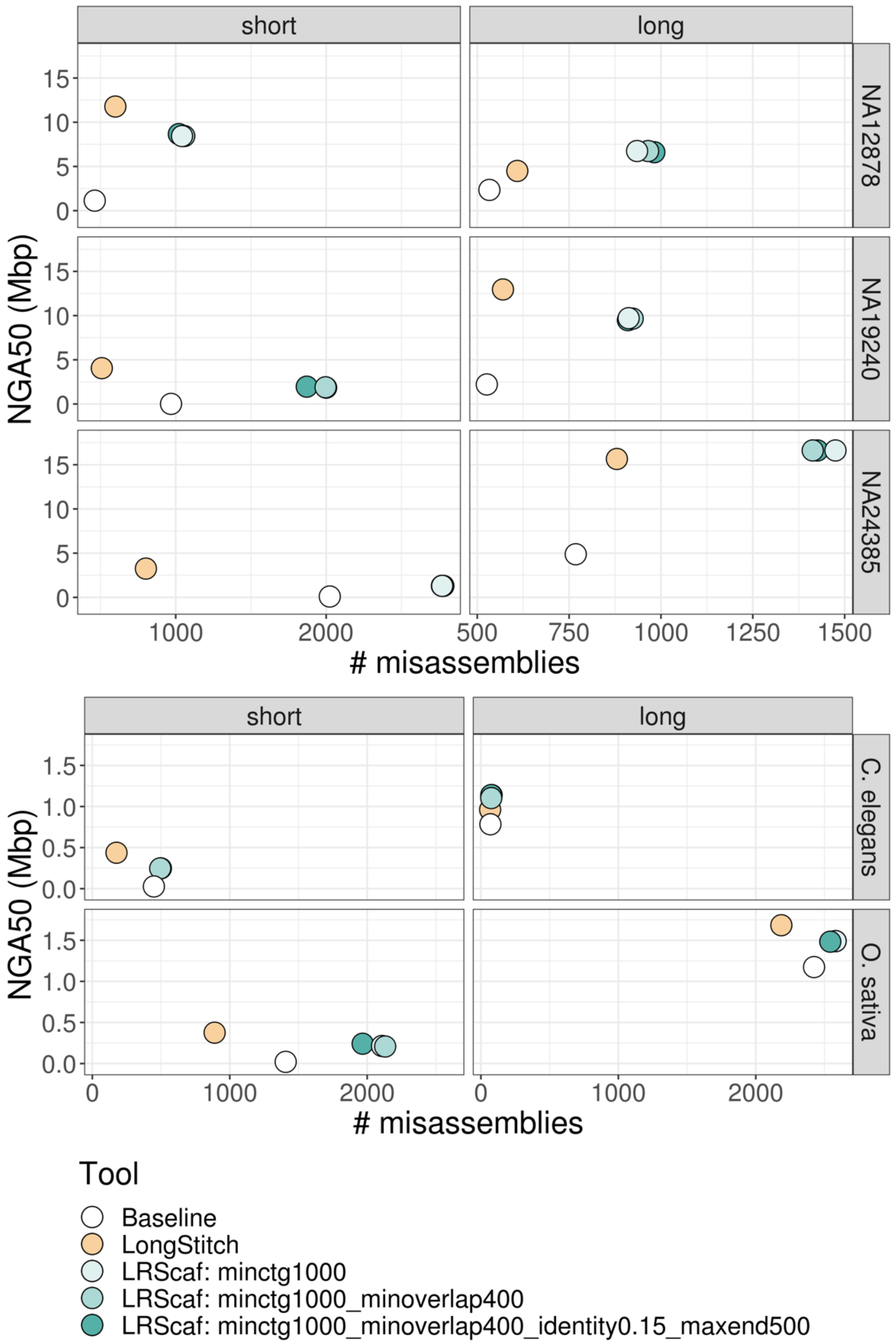

Figure S2. Contiguity and correctness statistics of assemblies improved using LRScaf with different parameter combinations and LongStitch. The LRScaf parameters tested are minctg1000: -micl 1000 -i 0.1; minctg1000_minoverlap400: -micl 1000 -mioll 400 -i 0.1; minctg1000_minoverlap400_identity0.15_maxend500: -micl 1000 -mioll 400 -i 0.15 -mxel 500 -mxohl 500.

Figure S3. Jupiter consistency plots showing the contiguity and correctness of LongStitch and LRScaf assemblies. Each (A) short-read ABySS assembly and (B) long-read Shasta assembly was improved with LongStitch (default steps) or LRScaf, and aligned to the human reference genome (GRCh38). The alignments are plotted as coloured ribbons in each Jupiter plot, with large-scale misassemblies evident as interrupting ribbons.

**(A)**	**short**	
**Individual**	**LongStitch**	**LRScaf**
**NA12878**	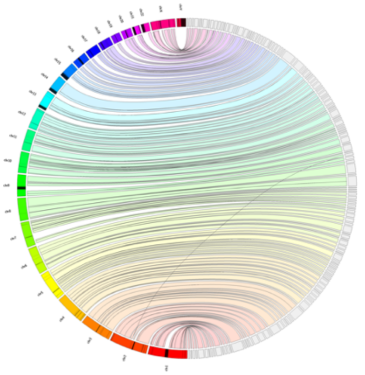	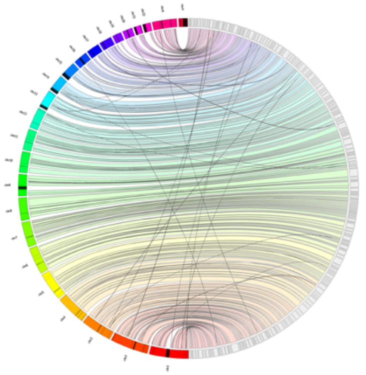
**NA19240**	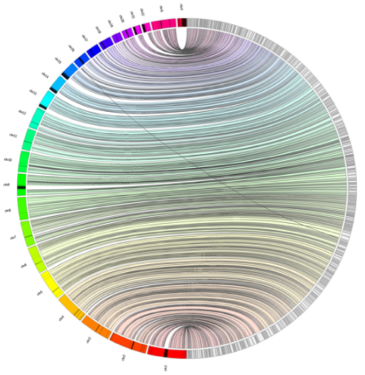	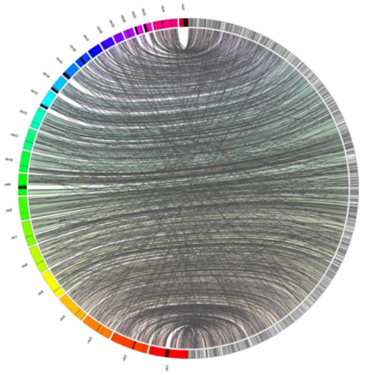
**NA24385**	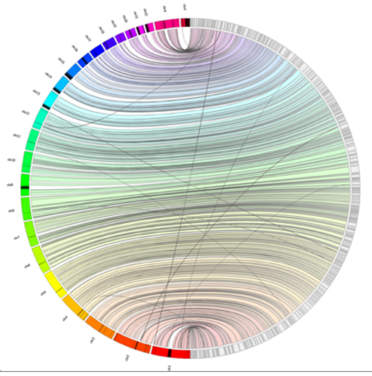	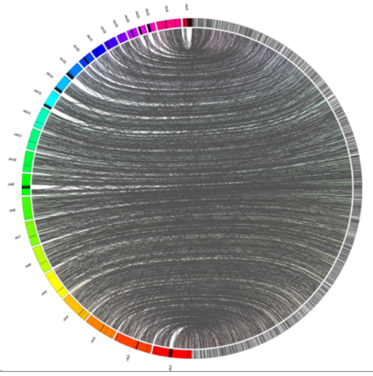

(B)	long	
**Individual**	**LongStitch**	**LRScaf**
**NA12878**	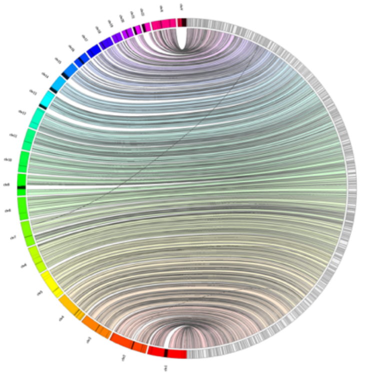	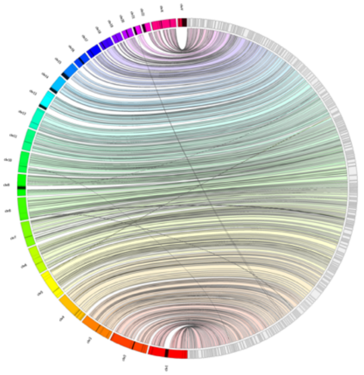
**NA19240**	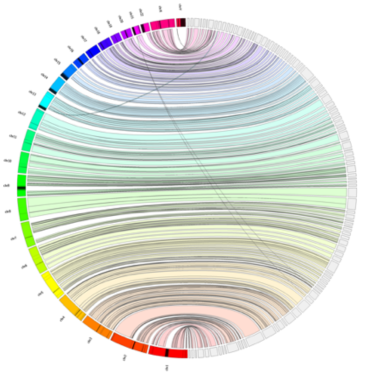	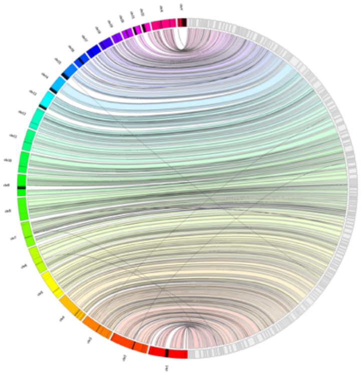
**NA24385**	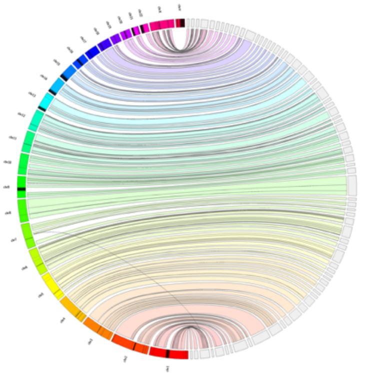	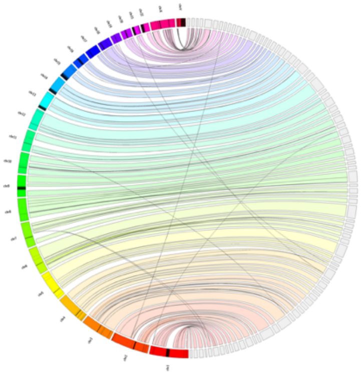

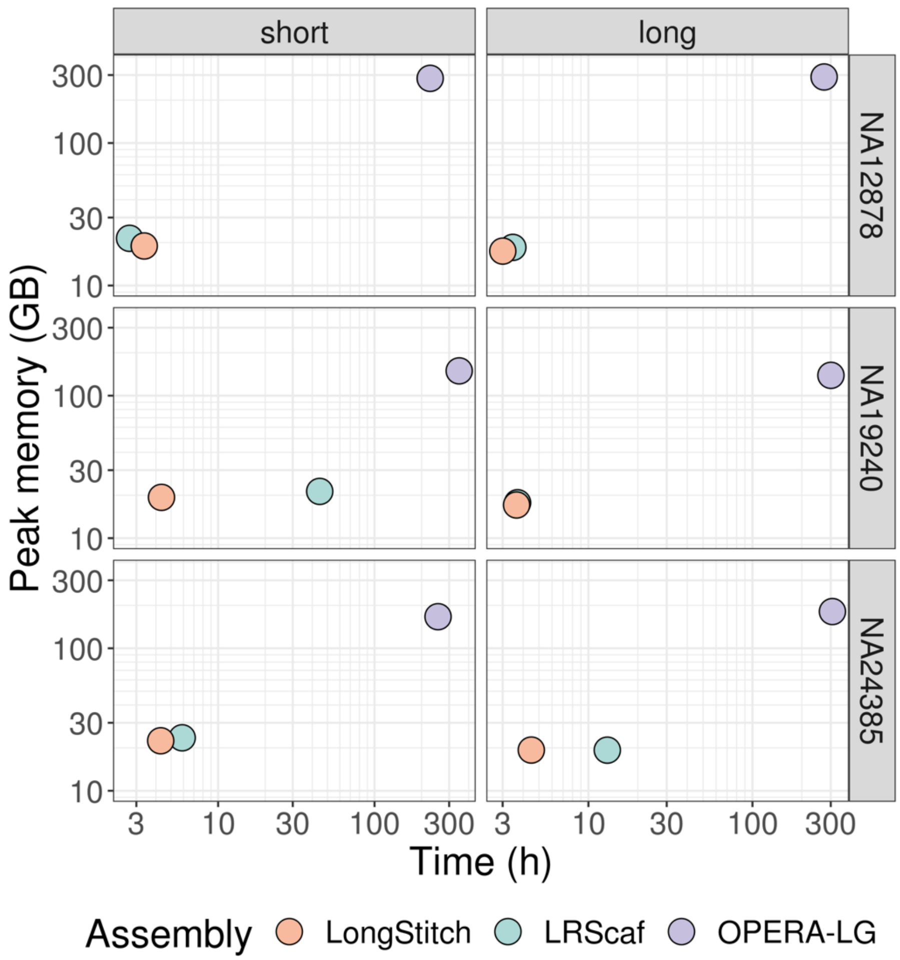

Figure S4. Benchmarking results of improving assemblies with LongStitch, LRScaf or OPERA-LG. For three human individuals, NA12878, NA19240 and NA24385, the wall-clock time and peak memory is shown for improving short-read ABySS assemblies and long-read Shasta assemblies using the default steps of LongStitch (up to ntLink) (orange), OPERA-LG (purple) or LRScaf (blue) on a log-log scale.

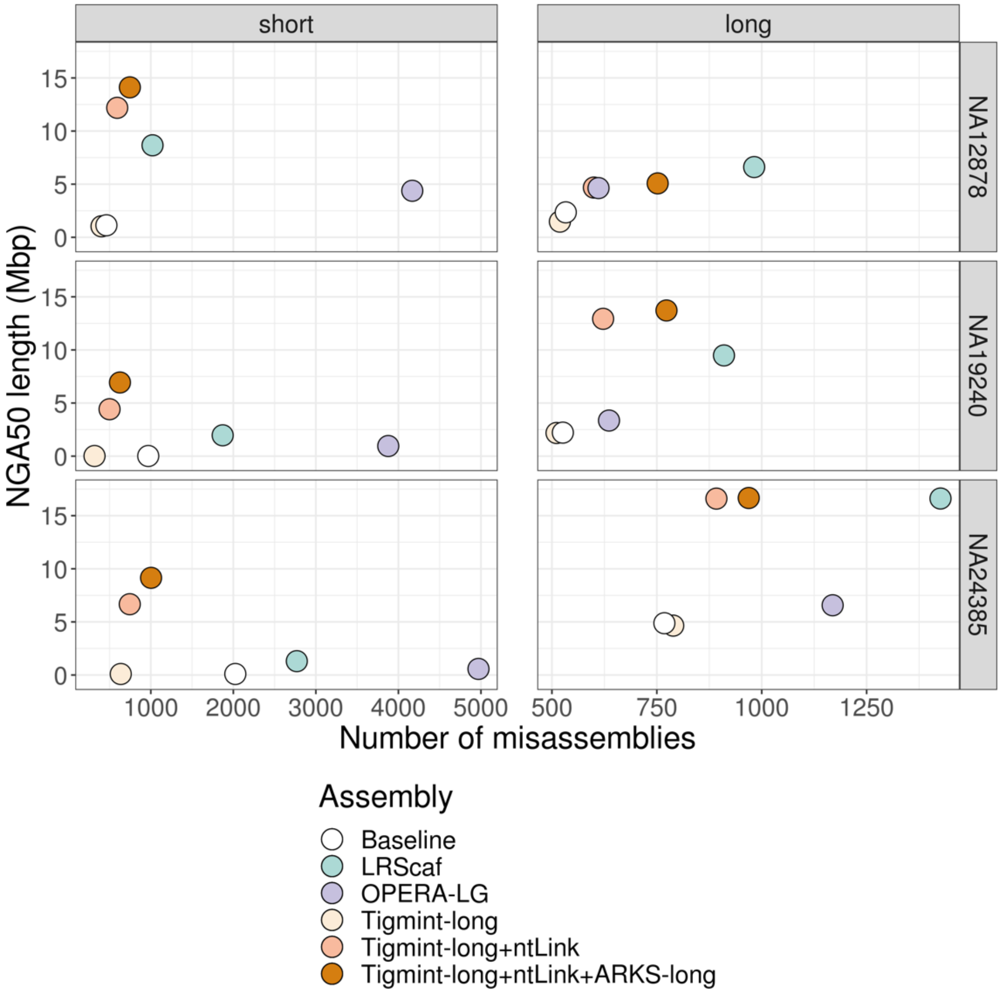

Figure S5. Contiguity and correctness results for running each step of the LongStitch pipeline, including the optional ARKS-long step, LRScaf and OPERA-LG on human assemblies. Each baseline assembly (white) was improved using LongStitch (orange), LRScaf (blue) and OPERA-LG (purple).

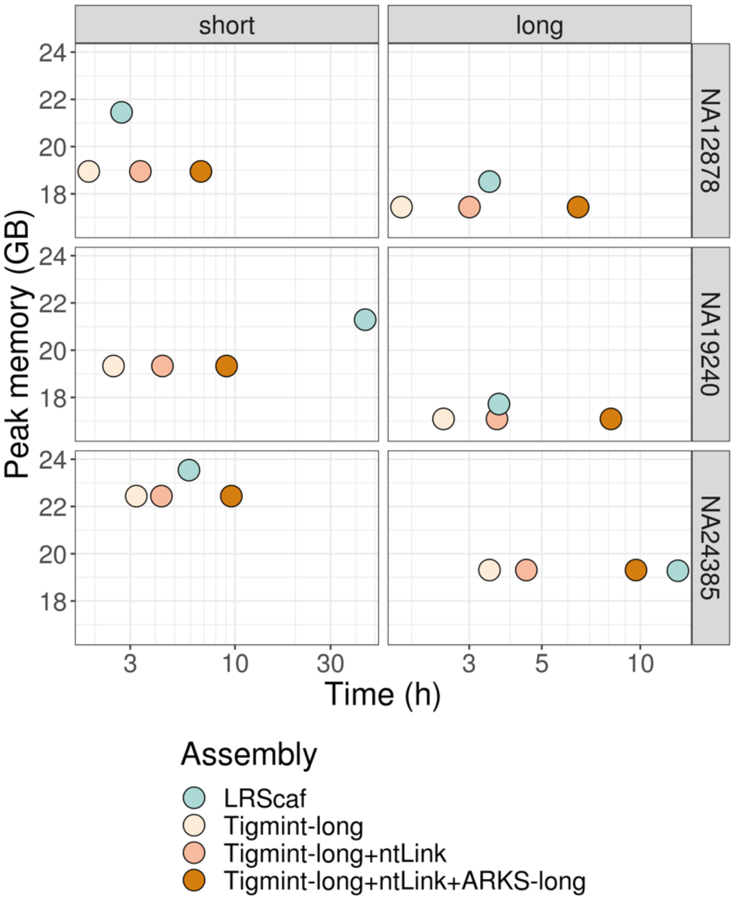

Figure S6. Benchmarking results for running LRScaf and the LongStitch pipeline including the optional ARKS-long step on human assemblies. All OPERA-LG runs on human data required over 9 days and 100GB of RAM to complete and are therefore not included in the plots.

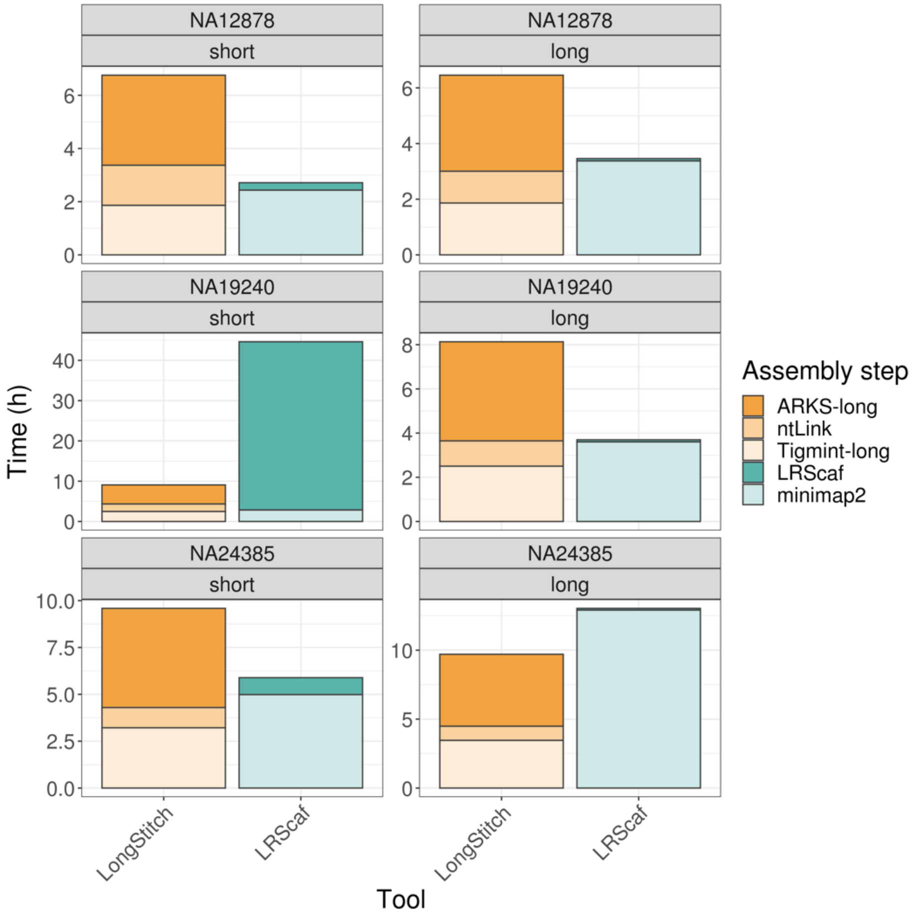

Figure S7. Time breakdown for each of the steps in LongStitch, including the optional ARKS-long step, and LRScaf. All OPERA-LG runs on human data required over 9 days to complete and therefore are not included in the plots.

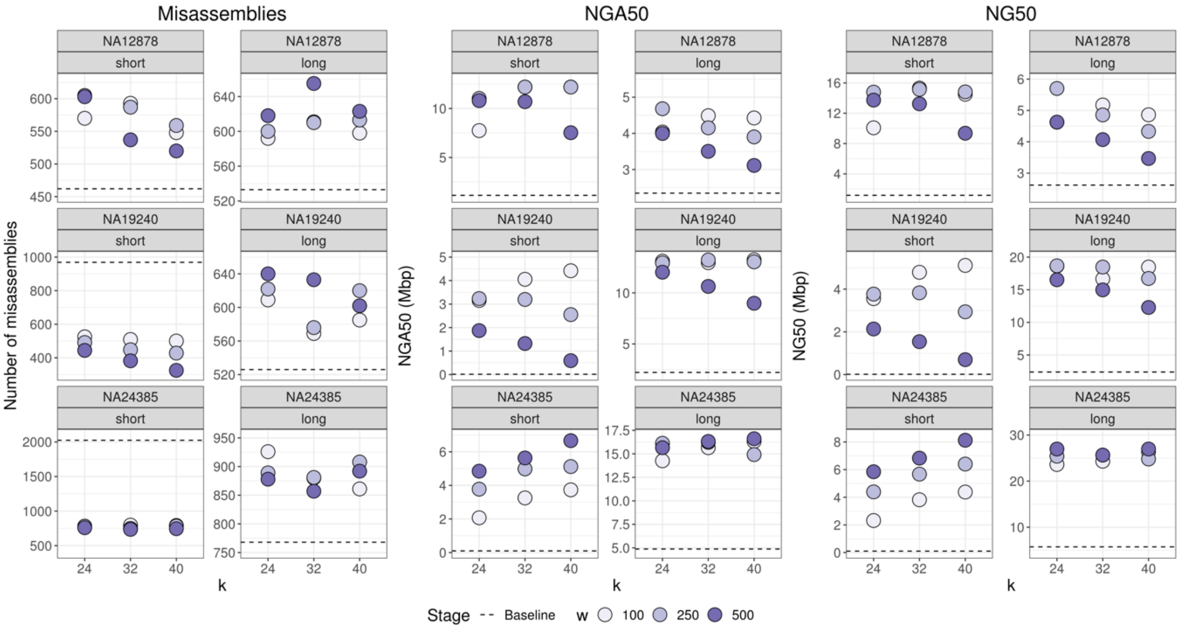

Figure S8. Contiguity and correctness results from sweeping on the ntLink *k* and *w* parameters for the default steps of LongStitch. The baseline assembly statistics are indicated with a horizontal dashed line.

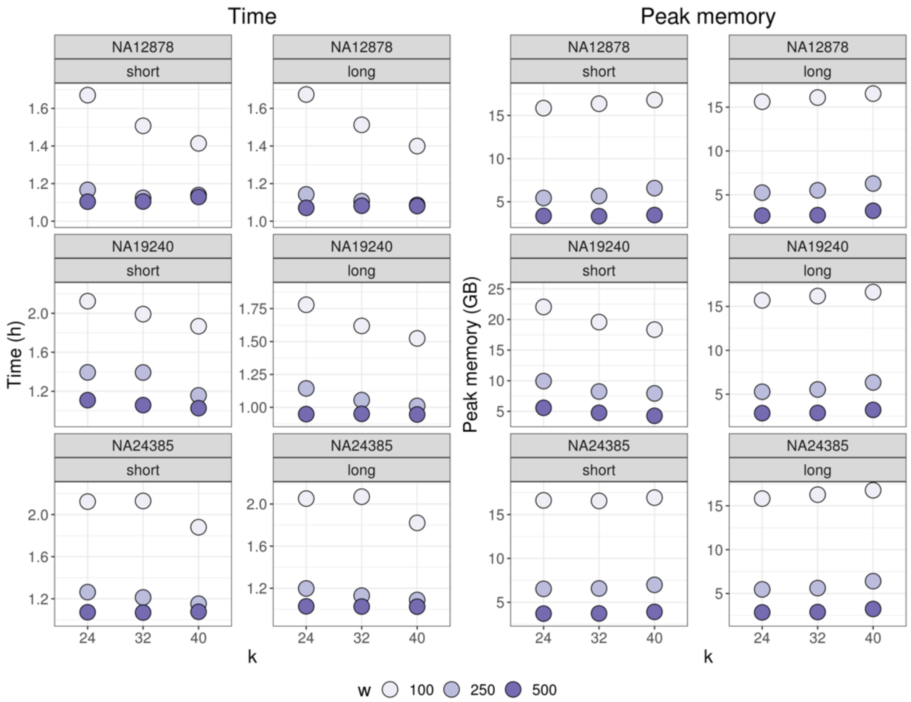

Figure S9. Benchmarking results from sweeping on the ntLink *k* and *w* parameters for the default steps of LongStitch.
